# Living Tissues by Design: The Rise of Hybrid Models in Biofabrication

**DOI:** 10.3390/jfb17030135

**Published:** 2026-03-10

**Authors:** Varvara Platania, Argyro Lamprou, Isaac Maximiliano Bugueno

**Affiliations:** 1 Orofacial Development and Regeneration, Institute of Oral Biology, Faculty of Medicine, Centre for Dental Medicine, University of Zurich, 8032 Zurich, Switzerland; argyro.lamprou@zzm.uzh.ch; 2 Institut de Génétique et de Biologie Moléculaire et Cellulaire (IGBMC), CNRS-UMR7104, INSERM U1258, Université de Strasbourg, 67404 Illkirch, France

**Keywords:** biofabrication, organoids, spheroids, self-organisation, 3D bioprinting, organ-on-chip, hybrid tissue models

## Abstract

Current in vitro tissue models struggle to recapitulate the structural, vascular, and mechanical complexity of human tissues, limiting their physiological relevance for disease modelling and preclinical testing. Self-organising three-dimensional cultures such as spheroids and organoids capture key aspects of cellular organisation and differentiation, but they commonly lack controlled geometry, perfusable vasculature, and reproducible mechanical microenvironments. Conversely, biofabrication strategies, such as three-dimensional (3D) bioprinting and organ-on-chip (OoC) microfluidic devices, offer spatial control, integrated perfusion, and dynamic mechanical stimulation, yet often fall short in recapitulating the full cellular diversity and self-organisation of native tissues. Notably, emerging hybrid approaches that embed self-organising biological units (e.g., organoids and spheroids) into engineered scaffolds or microfluidic platforms combine biological relevance, architectural fidelity, and functional control. Advances in bioink chemistry, sacrificial-printing vascularisation, and chip–organoid interfaces now enable perfusable, multicompartment tissues suitable for disease modelling and preclinical testing. This review highlights the most recent (2020–2025) progress in organoid vascularisation, bioprinting strategies for prevascularised constructs, and OoC integration, outlining remaining challenges and emphasising priorities for next-generation hybrid cellular and tissue models.

## 1. Introduction

The development of in vitro tissue models has been a cornerstone of biomedical research, enabling scientists to study human physiology, investigate disease mechanisms, and evaluate therapeutic candidates under controlled conditions [1]. Two-dimensional (2D) monolayer cultures have long served as the standard approach for cell-based studies, enabling human tissue modelling, supporting basic biological research, drug discovery, and regenerative medicine. However, these models often fail to replicate the complex architecture and dynamic microenvironment of native tissues, leading to discrepancies between in vitro and in vivo findings [2]. Early conceptual frameworks established that introducing a third dimension fundamentally alters cell behaviour, bridging the gap between conventional cell culture and living tissues [3,4]. Subsequent comparative studies systematically showed that three-dimensional (3D) culture environments reshape signalling, differentiation and drug responses relative to monolayers, underscoring the importance of dimensionality for physiological relevance [1].

The rise of 3D cell culture systems such as spheroids and organoids has emerged as more physiologically relevant in vitro models [4,5]. Spheroids are multicellular aggregates, usually formed from differentiated or cancerous cell lines, which offer a simplified 3D architecture for studying cell–cell interactions, metabolic gradients and tumour-like microenvironments [3,6]. In contrast, organoids are 3D cultures derived from stem cells, generated from pluripotent stem cells or adult stem cells residing in tissues, that self-organise within an extracellular matrix and reproduce key aspects of native tissue architecture, cellular diversity, and developmental programmes [7,8,9,10]. The formation of organoids relies on intrinsic morphogenetic processes supported by defined niche factors, which distinguishes these systems from spheroids and other artificial 3D aggregates [11,12]. These self-organising systems often remain limited by variable morphology and restricted size (due to diffusion limits) [13]. Additionally, they lack perfusable vasculature and mechanical inputs that underlie tissue maturation and pathology [13]. Recent studies emphasise vascularisation and microenvironmental control as the critical challenges to move organoids from descriptive models toward predictive, transplantable tissues [14].

Concurrently, biofabrication technologies such as 3D bioprinting and organ-on-chip systems have matured, offering tools for spatial patterning, mechanical stimulation, and integration of complex tissue compartments [15,16]. These engineered platforms allow precise control over the physical and biochemical microenvironment, addressing many of the constraints of traditional models. Organ-on-chip systems, in particular, have demonstrated how mechanical cues (e.g., cyclical stretch or shear) and microfluidic perfusion dramatically change cellular behaviour [17,18], drug uptake [18,19], and barrier functions [17,18,19]. The combination of three-dimensional tissue organisation with controlled perfusion and mechanical cues enables the reconstitution of organ-level functions in vitro, marking a conceptual shift from static cultures toward dynamic, physiologically active models [19]. These capabilities can complement organoid biology when combined thoughtfully [15].

As biological models increased in cellular complexity and scale, traditional bottom-up self-organisation alone proved insufficient to achieve reproducible tissue architecture, controlled spatial patterning, and integration of vascular and stromal compartments. Bioprinting emerged in response to this gap, providing a means to externally impose geometry, cellular organisation and material heterogeneity while remaining compatible with living cells and dynamic remodelling [20,21]. By enabling the spatially resolved deposition of multiple cell types, biomaterials and biochemical cues, bioprinting offers a level of architectural and microenvironmental control that is difficult to achieve with self-assembled systems alone [21,22]. This capability has positioned bioprinting as a central biofabrication process for constructing perfusable, multicompartment tissues and for interfacing biological self-organisation with engineered structure, a prerequisite for scaling tissue models toward functional maturity and translational relevance [23,24].

We argue that the most promising path forward is interactive integration and rational design of engineered microenvironments guided by biological self-organisation, supported by computational design and machine-learning optimisation, to accelerate maturation and reproducibility [25,26]. Instead of replacing organoids and spheroids, hybrid systems hold the potential to unify biological complexity with engineering precision, facilitating more robust and physiologically accurate in vitro models.

**Figure 1:**
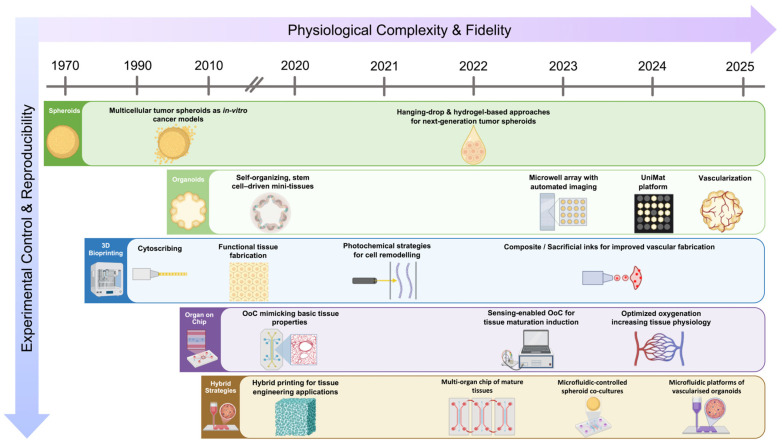
Schematic overview of the historical and technological evolution of biofabricated in vitro models. The timeline (1970–2025, indicated by the upper grey axis) is divided in major biofabricated systems (spheroids in dark green, organoids in light green, 3D bioprinting in blue, organ-on-chip platforms in purple, and hybrid strategies in brown). Landmark advances or approaches within each system have been mentioned along the axis. The two arrows highlight the trends across the various systems: the increase in physiological complexity and fidelity (purple horizontal arrow) and the increase in experimental control and reproducibility (blue vertical arrow). The information depicted has been extracted from the following original studies: [4,22,24,27,28,29,30,31,32,33,34,35,36,37,38,39,40,41,42,43,44,45,46,47,48,49,50,51,52,53]. Created in BioRender.

Unlike existing reviews that focus on individual platforms in isolation, this review provides a unified and critical synthesis of recent advances (2020–2025) in spheroid- and organoid-based systems, 3D bioprinting, and organ-on-chip technologies, framed through the lens of hybrid biofabrication. By explicitly analysing how engineered boundary conditions, perfusion strategies, and embedded sensing interact with biological self-organisation, we propose a conceptual framework that positions hybridisation as a design paradigm rather than a collection of tools and identify key translational bottlenecks and priorities for next-generation in vitro tissue models (Figure 1).

## 2. Traditional 3D Culture Models: From Spheroids to Organoids

### 2.1. Spheroids: Simplified, Scalable, but Limited in Architecture and Perfusion

Multicellular spheroids represent the foundational model of 3D cell culture, providing a minimal yet physiologically relevant architecture. They were first systematically characterised as a 3D tumour model. In 1971, Sutherland et al. demonstrated in vitro that tumour cells cultured in suspension form spherical aggregates with outer proliferative layers and inner hypoxic or necrotic cores resembling avascular tumours [4]. This organisation provided a valuable alternative to 2D monolayers for cancer research and radiobiology in vitro.

Shortly after, the ability of human tumour specimens to form spheroids directly from surgical material was shown, establishing their clinical relevance [27]. Canonical fabrication techniques include hanging drop, liquid overlay/low adhesion plates, and spinner or rotary suspension systems, which leverage spontaneous aggregation and hydrodynamic conditions to form uniform 3D aggregates. On the opposite side, modern engineering adaptations employ microfluidics and patterned wells for enhanced control, throughput, and size uniformity [28].

For instance, Rodoplu et al. recently developed a microfluidic hanging-drop platform that allows co-culture of distinct cell types within precisely defined droplets, producing uniform spheroids while maintaining dynamic nutrient exchange [29].

Similarly, Khan et al. used droplet-based microfluidics and thiol–acrylate hydrogels to encapsulate cells in tuneable microenvironments [30], demonstrating reproducible spheroid size and enhanced viability compared with conventional batch methods.

Complementary work by Wongpakham et al. optimised pyramidal microwell geometries to promote rapid cell aggregation and homogenous spheroid formation, underscoring how microscale design directly dictates tissue organisation [31].

#### 2.1.1. Strategies Developed Between 2020 and 2025

These fabrication advances have paved the way for a new generation of engineered and perfused spheroid systems. Fang et al. reviewed how microfluidic integration enables controlled perfusion, mechanical stimulation (shear stress, compression), and real-time optical monitoring of spheroids and organoids derived from tumour, hepatic, intestinal, cardiac, and neural lineages [32]. Their analysis shows that perfused spheroid-on-chip platforms better preserve tissue polarity, metabolic activity, and drug responsiveness than static cultures, thereby extending viability from days to several weeks and enabling longitudinal functional studies.

Building on this concept, Quintard et al. developed a vascularised microfluidic platform in which tumour spheroids, pancreatic islet spheroids, and blood-vessel organoids derived from human pluripotent stem cells were embedded adjacent to endothelialised microchannels formed by primary human endothelial cells [33]. These channels established functional perfusion and inosculated with the surrounding tissue, creating a hybrid biological-engineered vasculature that significantly reduced hypoxia, improved nutrient transport, and enhanced tissue-specific functional readouts, including insulin secretion and metabolic activity. This work demonstrated that engineered perfusion could synergise with biological self-organisation to generate more physiologically faithful microtissues suitable for disease modelling and drug testing.

To address reproducibility and quantitative scalability, Dornhof et al. introduced a bioprinting-assisted automated deposition system that places individual cancer cell spheroids (derived from human tumour cell lines) into oxygen-sensor microelectrode wells with micrometre precision [26]. Their platform enables real-time monitoring of oxygen consumption and metabolic gradients within each spheroid, transforming spheroids from passive 3D models into quantifiable biological units that can be spatially organised, continuously monitored, and statistically compared. Together, these approaches illustrate how spheroids are no longer endpoint models but are becoming modular, vascularised, and sensor-integrated building blocks for next-generation hybrid tissue systems (Table 1).

#### 2.1.2. Current Limitations

Nevertheless, intrinsic biophysical limits remain. Debruyne et al. used multimodal oxygen probes to map intra-spheroid gradients, revealing pronounced hypoxia and necrosis beyond 200 μm from the periphery [35]. Such diffusion-limited microenvironments, together with the absence of vasculature and stromal complexity, undermine physiological fidelity.

Reviews by Nayak et al. [36] and Liu et al. [37] highlight how these factors reduce the predictive accuracy of spheroid-based drug assays and motivate integration with perfused microfluidic or printed scaffolds. Spheroid models more accurately capture three-dimensional cell–cell and cell–matrix interactions, as well as nutrient and metabolic gradients, than conventional 2D cultures. However, they lack key features of native tissues, including perfused vasculature, dynamic transport of nutrients and drugs, and mechanical cues. These limitations lead to heterogeneous drug penetration, non-physiological hypoxic or quiescent regions, and altered metabolic and stress responses. As a result, the predictive accuracy of spheroid-based drug assays remains limited when compared with in vivo outcomes. Both reviews therefore advocate integrating spheroids with perfused microfluidic systems or engineered scaffolds to restore controlled mass transport, vascular interfaces, and microenvironmental regulation.

Thus, spheroids now serve not as endpoints but as biological building blocks for hybrid, vascularised, and mechanically dynamic tissue models that bridge simplicity with physiological relevance.

Collectively, these advances move spheroids from being static, simplified 3D assays to modular biological units that can be quantitatively manufactured, spatially organised, and functionally interrogated. While spheroids alone cannot replicate the full structural, vascular, and mechanical complexity of native tissues, their robustness, scalability, and intrinsic ability to generate physiological gradients make them particularly well suited as building blocks in more complex biofabricated systems. In this context, spheroids are increasingly being used not as stand-alone tissue substitutes but as reproducible building blocks that can be integrated into artificial scaffolds, perfused microfluidic platforms or combined with stem cell-derived systems to progressively approximate tissue-level organisation and function.

### 2.2. Organoids: Powerful Self-Organisation, Constrained by Scale, Reproducibility, and Microenvironmental Control

Organoids are three-dimensional, self-organising assemblies derived from pluripotent or adult stem cells that capture key features of organ morphogenesis, cell differentiation, and specialised tissue function that 2D cultures and simple spheroids cannot reproduce. The modern organoid field was established with the seminal discovery that single adult LGR5^+^ intestinal stem cells can self-organise into long-lived, functional crypt–villus structures in 3D extracellular matrix cultures when provided with defined niche signals, demonstrating for the first time that complex tissue architecture can emerge in vitro without mesenchymal support [7]. This breakthrough distinguished organoids from earlier 3D aggregates and embryoid bodies and laid the foundation for a rapidly expanding class of self-organising stem-cell-derived tissues. Organoids are now broadly categorised based on their cellular origin and developmental strategy into adult stem cell–derived epithelial organoids, pluripotent stem cell–derived organoids that recapitulate developmental programmes, and patient-derived tumour organoids for disease modelling and precision medicine [38].

The Nature Reviews Methods Primers article by Zhao et al. outlined the methodological foundation of organoid generation, detailing how cell origin, matrix composition, and growth-factor gradients jointly define organoid identity and architecture [11]. They emphasised that intrinsic self-organisation recapitulates developmental patterning but also introduces variability in size, geometry, and cellular composition. These are limitations that restrict quantitative comparison and translational use; thus, engineering frameworks are increasingly applied to control the external environment of organoids.

#### 2.2.1. Strategies Developed Between 2020 and 2025

Recent engineering-driven organoid platforms have explicitly targeted tissue specificity and maturation. Kim et al. developed the UniMat system to generate uniform kidney and intestinal organoids from human pluripotent stem cells, reporting enhanced nephron segmentation, epithelial polarisation, and expression of maturation markers compared to conventional Matrigel cultures [39]. By tuning nutrient flux and boundary curvature, UniMat cultures produced kidney and intestinal organoids of nearly identical diameters and exhibited higher expression of maturation markers than conventional Matrigel droplets.

Recent work has extended organotypic 3D culture principles to developmental interfaces involving reciprocal epithelial–mesenchymal interactions. Jerbaka et al. established an organotypic bilayer model mimicking early craniofacial tooth development, combining epithelial cells with neural crest–derived ectomesenchyme, thereby restoring reciprocal signalling and tissue polarity absent from homogeneous spheroids [40]. By preserving bilayer organisation and reciprocal tissue crosstalk, this system captures key developmental mechanisms that are lost in conventional monolayers or homogeneous spheroids.

Likewise, Sockell et al. applied automated longitudinal imaging to intestinal and hepatic adult stem cell–derived organoids, enabling real-time correlation between morphological growth trajectories and differentiation outcomes [41]. They reported a two-fold reduction in morphological variance and demonstrated that real-time phenotyping could predict differentiation trajectories. Together, these studies illustrate a shift from descriptive, heterogeneous organoid cultures to quantitatively engineered reproducible systems.

Further integration with microfluidic and mechanical technologies has propelled organoids toward dynamic, perfused constructs. Wang et al. reviewed recent organoid-on-chip designs that deliver continuous perfusion, shear stress, and cyclic stretch, showing how mechanical conditioning enhances barrier integrity, epithelial polarity, and electrophysiological activity [54].

Toward dynamic systems, Quintard et al. embedded pancreatic islet spheroids, hepatic organoids, and vascular organoids derived from human iPSCs into endothelialised microfluidic chips, achieving perfusion-dependent improvements in oxygenation, metabolic activity, and hormone secretion [33]. Their vascularised chips maintained stable perfusion for over two weeks and demonstrated improved oxygenation and functional readouts compared with static controls.

Similarly, Nwokoye et al. demonstrated that combining brain, hepatic, and cardiac organoids with endothelial progenitor cells and microfabricated channels yields perfusable hybrid vascular networks that better replicate tissue-scale transport and maturation [55].

Collectively, these studies show that external microengineering can guide self-organisation toward quantitatively engineered reproducible systems (Table 2).

#### 2.2.2. Current Limitations

Despite their progress, organoids remain imperfect surrogates of native tissues. Maggiore et al. used a genetically inducible endothelial niche to vascularise kidney organoids, achieving organised microvessels. However, they reported incomplete perfusion and persistent foetal-stage transcriptional profiles [57].

Similarly, Werschler et al. analysed multiple vascularisation strategies and observed that premature endothelial introduction can perturb developmental patterning, underscoring the delicate balance between guided maturation and developmental staging [58].

Moreover, long-term perfused cultures demand materials compatible with sustained flow and optical interrogation; Li et al. reported that device material heterogeneity and manual assembly contribute significantly to variability across laboratories [42]. These limitations highlight the balance between biological spontaneity and engineering precision: efforts to impose structure or perfusion can inadvertently alter the very morphogenetic programmes that give organoids their authenticity.

Current consensus across these studies is that organoid systems benefit most when self-organisation is guided. In this framework, engineering defines the boundaries, nutrient transport, and mechanical cues, while the cells’ own biological programme drives their differentiation. The emerging convergence of scalable fabrication [39,41], perfusable vascular design [33,55], and integrated sensing platforms points toward physiologically faithful and experimentally reproducible organoids. Yet, to achieve adult-like function and enable cross-tissue integration, future biofabrication approaches will need to combine organoid biology with 3D printing, microfluidics, and computational design principles—a hybrid strategy explored in the following sections.

### 2.3. Bridging the Gap: Toward Engineered Complexity

A unifying theme that has emerged from 2020 to 2025 is that neither self-organisation nor top-down engineering alone suffices to recapitulate tissue-level physiology; instead, hybridising organoids or spheroids with engineered microenvironments preserves developmental programmes while supplying the geometry, perfusion and mechanical cues they lack. Microfluidic vascularisation demonstrates this synergy: embedding organoids adjacent to endothelialised channels yields sustained perfusion, improved oxygenation and accelerated functional maturation compared to static culture [33]. In parallel, engineering advances have produced capillary networks capable of perfusing multi-millimetre tissues, expanding the limits of oxygen and nutrient transport that underlie necrotic cores in larger constructs [43]. Complementary biofabrication efforts now use printing and hybrid deposition to place preformed spheroids or organoid aggregates into defined matrices or support baths, combining positional control with innate self-assembly to reduce heterogeneity and enable downstream readouts [55]. Reviews of vascularisation strategies and organoid–chip integration emphasise that the most productive pathways couple engineered channels and controlled flows with biological angiogenesis and niche signals, rather than imposing rigid templates that disrupt morphogenesis [55]. Together, these studies set the stage for Section 3: 3D bioprinting and organ-on-chip platforms that translate hybrid principles into reproducible, perfusable model systems for disease modelling and preclinical testing.

## 3. Biofabrication Approaches

### 3.1. 3D Bioprinting: Enabling Geometry, Perfusion, and Multi-Material Tissues

3D bioprinting has matured from a proof-of-concept tool into a central technology for assembling multicellular architectures with controlled geometry, mechanical gradients, and perfusable vasculature. Unlike self-organising organoids, which rely on intrinsic morphogenesis, bioprinting offers external control over spatial patterning and integration of vascular or stromal compartments. Over the last five years, progress has focused on bridging biological complexity with engineered structure to produce tissues that are both architecturally precise and biologically functional [25].

#### 3.1.1. Evolution of 3D Bioprinting Methods

Bioprinting originated from early attempts to spatially control cell placement using engineering-inspired patterning techniques, most notably Klebe’s 1988 demonstration of “cytoscribing”, which established that living cells could be positioned with micrometric precision to guide tissue assembly [59]. The field advanced decisively in the early 2000s when Boland, Mironov, and colleagues adapted inkjet printing technologies for biological use, demonstrating layer-by-layer deposition of viable mammalian cells and introducing the concept of computer-aided “organ printing” as a scalable tissue fabrication strategy [21,60]. These studies established cell viability and post-print self-organisation as central principles, triggering rapid diversification of printing modalities. During the subsequent decade, bioprinting methods were systematically classified into inkjet-based, extrusion-based, and laser-assisted approaches, each offering distinct advantages in resolution, cell density, and material compatibility [20]. By the mid-2010s, the field shifted from simple patterning toward functional tissue fabrication, emphasising vascularisation, multi-material printing, and integration with bioreactors and microfluidic systems. Contemporary bioprinting now converges with stem cell biology, organoid technology, and organ-on-chip platforms, positioning it as a core biofabrication technology for generating perfusable, multicellular, and increasingly physiologically relevant tissue models [23,44].

Recent work by Leung et al. [15] and Wang et al. [54] emphasised that integrating biochemical and mechanical tunability enables the printing of tissues that better mimic native viscoelasticity while remaining compatible with organ-on-chip interfaces. Likewise, a 2024 *Biofabrication* study introduced bioinks that incorporate angiogenic peptides and degradable crosslinkers, achieving improved vascular infiltration compared to inert hydrogels [22]. These hybrid inks mark a transition from “printable gels” toward biofunctional microenvironments that guide morphogenesis post-printing.

#### 3.1.2. Strategies Developed Between 2020 and 2025 That Enable Hybrid Functionality

(a) Sacrificial-ink vascularisation

Sacrificial (or fugitive) templating remains a practical route to produce hierarchical, perfusable channels inside cell-dense matrices. Quintard et al. combined sacrificial gelatine channels with organoid-laden matrices inside microfluidic chips, achieving continuous perfusion for more than 14 days and enhanced oxygenation relative to static controls. Their platform directly illustrates how printed networks can be combined with living microtissues, a key theme linking bioprinting to organ-on-chip integration [33].

Malkani et al., in 2025 [45], demonstrated systematic use of fugitive templates, such as Pluronic, gelatine and carbohydrate-glass variants, and quantified how template geometry and removal strategy affect channel fidelity and endothelial seeding efficiency. Their study demonstrated that optimised sacrificial patterns permit rapid vascularisation and stable perfusion in millimetre-scale constructs [45].

Complementary engineering of the surrounding support medium reduces gravitational collapse during casting and preserves channel geometry after sacrificial removal, enabling larger, more complex vascular trees. Budharaju et al. in 2024 validated embedded printing of fugitive templates in support baths and reported improved channel fidelity and cell viability in dense parenchymal matrices [61].

(b) Coaxial extrusion

Coaxial extrusion produces continuous, lumenised filaments in a single step; an attractive route to fabricate tubular vessels with concentric layers. Li et al. developed concentric-nozzle printing of collagen–alginate hybrids that immediately form tubular microvessels, which can be endothelialised and perfused without post-processing [42]. Coaxial strategies enable scalable fabrication of hierarchical vascular trees that sustain centimetre-scale tissues, which cannot be achieved solely by spontaneous angiogenesis.

In 2022, Bosch-Rué et al. used support-free coaxial extrusion of high-concentration collagen to print tubular tissue-engineered blood vessels with physiologically relevant burst pressures and high endothelial viability, demonstrating that pure protein bioinks can form mechanically robust, perfusable tubules without ancillary scaffolds [46].

Finally, recent optimisation studies have clarified print parameters. For instance, Rahman et al. measured how inner-nozzle diameter and flow ratios govern filament gelation and mechanical strength, providing practical design rules for reproducible coaxial fabrication [62].

(c) Spheroid- or organoid-assisted bioprinting

Recently, bioprinting strategies that use preformed spheroids or organoids as modular building blocks have emerged as an alternative to the deposition of dispersed single cells. Dornhof et al. demonstrated the automated placement of preformed tumour spheroids into oxygen-sensor microelectrode wells, achieving high spatial precision together with real-time metabolic monitoring [26].

Additionally, Huang et al. reviewed how printing stem-cell-derived organoids as “bio-building blocks” accelerates tissue maturation by maintaining intrinsic cell–cell signalling while benefiting from reproducible architecture [25].

Recent strategies increasingly employ preformed microtissues as biological building blocks. Kim et al. developed a high-throughput bioprinting platform (HITS-Bio) to spatially pattern tumour spheroids composed of human breast and glioblastoma cells, preserving paracrine signalling while enabling scalable tissue assembly and oxygen monitoring [34].

In 2023, Roth et al. introduced the SPOT (Spatially Patterned Organoid Transfer) platform to pattern human iPSC-derived cortical and spinal cord organoids, enabling reproducible neurodevelopmental assembloids with defined spatial organisation and synaptic connectivity [43]. These modular approaches accelerate tissue-level maturation by maintaining intrinsic cell–cell interactions while benefiting from externally imposed architectural control [56].

These studies illustrate the convergence between biological self-organisation and technical precision, demonstrating how modular microtissues can be assembled into higher-order architectures that more accurately replicate tissue development and physiology.

(d) Light-based printing

Vat photopolymerisation modalities (SLA/DLP) have advanced to pattern soft, cell-compatible hydrogels at high resolution. Debruyne et al. optimised photopolymerisation kinetics to reduce phototoxicity, enabling voxel-scale patterning (<50 μm) within soft hydrogels. Such advances permit precise microchannel formation inside cell-dense constructs, extending the functional reach of hybrid inks [35].

In 2023, Lin et al. used gelatine-norbornene (GelNB) in DLP bioprinting to fabricate perfusable microchannels and soft cell-laden architectures with good viability, showing that photochemical formulations can produce low-stiffness constructs amenable to cell remodelling [47].

Meanwhile, from 2021 to 2023, different research groups optimised visible-light photoinitiators and exposure regimes (e.g., eosin-based or ruthenium systems) to reduce phototoxicity while achieving rapid voxel curing, providing practical photochemistry that preserves cell viability during high-resolution patterning [63,64].

(e) Mechanical and perfusion conditioning

Post-print conditioning through controlled flow, cyclic stretch, and electrical pacing markedly accelerates maturation. Fang et al. reported that cyclic flow within printed vascularised constructs increases endothelial barrier integrity and parenchymal differentiation, underscoring the need to integrate biomechanical stimuli into printed tissues [32].

In 2023, Mitchell et al. characterised vascular bioreactors where pressure-driven pulsatile flow strengthened engineered vessel walls and improved endothelial barrier function [48], while Putame et al. demonstrated tuneable stretch bioreactors that promote structural and functional maturation across tissue types [65].

The same year, Komosa et al. reported that perfusion applied immediately after printing (intermittent or continuous regimes) enhances cell density and survival in printed chambers. This was in line with several 3D-printed bioreactor studies that reported increased parenchymal differentiation and reduced necrosis [66]. The limitations of conventional 3D bioprinting and hybrid strategies, developed from 2020 to 2025, enabled complex tissue integration presented in Table 3.

#### 3.1.3. Practical Outcomes and Remaining Challenges

Collectively, these innovations have produced printed tissues several millimetres thick that sustain perfusion and exhibit adult-like functions such as barrier transport, contractility, and electrophysiology. Yet, major challenges remain. Even the most advanced constructs struggle to reproduce capillary-scale (<10 µm) vasculature at biologically relevant speed or achieve functional anastomosis with host vessels [57]. Additionally, print-resolution versus speed trade-offs and mechanical mismatch between printed scaffolds and soft parenchyma continue to limit long-term stability.

Ongoing work in rheologically tuneable hybrid inks [22] and automated spheroid assembly [26] suggests a coming synthesis: bioprinting as the structural arm of hybrid biofabrication, where self-organised biological units are guided by engineered geometry, perfusion, and mechanical feedback. This foundation directly informs the next section on organ-on-chip technologies, which extend these printed architectures into dynamic microfluidic environments for long-term culture, multi-tissue coupling, and high-content functional analysis for translational applications.

A comparative assessment of the relative strengths and limitations of the major 3D culture and biofabrication platforms discussed in this review, across key structural, functional, and translational properties, is summarised in Table 4.

### 3.2. Organ-on-Chip Technologies: Microengineered Platforms for Dynamic Physiology

OoC platforms bring microfluidic control, mechanical actuation, and integrated sensing to engineered tissues, providing temporal and spatial cues that profoundly alter cell behaviour compared with static cultures. This class of systems has evolved from single-tissue barrier models into modular, vascularised, and multi-organ networks that enable long-term culture, controlled perfusion, and high-content functional readouts that directly complement biofabrication strategies for hybrid tissues [15].

#### 3.2.1. Evolution

Early OoC studies emphasised simple laminar-flow chambers and stretchable membranes to model barriers (e.g., lung or gut), demonstrating how shear and cyclic strain change physiology in ways impossible to reproduce in static wells. Building on these proof-of-concepts, recent studies prioritised vascular integration and organoid compatibility. For instance, Quintard et al. fabricated a user-friendly microfluidic platform that embeds and vascularises diverse 3D tissues (spheroids, blood-vessel organoids, and pancreatic islets), achieving stable perfusion and improved maturation over more than 14 days [33].

Concurrently, reviews and method papers showed that chips can constrain boundary conditions, such as flow, oxygen gradients, and mechanical loading, to reduce organoid heterogeneity and accelerate maturation [33]. Wang and Qin surveyed advances in organoids-on-chips and highlighted applications in developmental modelling and disease, stressing that microfluidic control is often necessary to translate organoids from descriptive to predictive platforms [49].

Recent work also highlights the rapid progress of microfluidics-based microphysiological systems, where organ-on-a-chip platforms are being tailored specifically to dental, oral, and craniofacial tissues, providing highly controlled dynamic microenvironments that closely recapitulate structural and mechanical features of the in vivo oral environment [50].

#### 3.2.2. Strategies Developed Between 2020 and 2025 That Enable Hybrid Functionality

(a) Perfusable vascular networks on chip

Currently, studies are shifting towards coupling engineered channels with biological angiogenesis by printing or moulding microchannels. This provides immediate perfusion while embedded endothelial cells or angiogenic factors promote vessel maturation and inosculation.

Hybrid OoC platforms increasingly model organ-specific physiology under flow. Quintard et al. generated vascularised chips integrating pancreatic, hepatic, and vascular organoids from human iPSCs and primary endothelial cells, achieving sustained intravascular perfusion and tissue-specific functional outputs such as insulin secretion and metabolic regulation. They directly measured improved oxygenation and organoid maturation under flow, demonstrating that engineered channels can immediately relieve hypoxia while connecting to tissue vasculature on-chip [33].

The same year, Debruyne et al. introduced Near-Infrared (NIR) ratiometric oxygen nanosensors for live mapping of oxygen gradients inside multicellular spheroids, providing a quantitative tool to validate how printed/perfused networks change local O_2_ microenvironments [35]. Such sensors are indispensable for proving that printed channels and chip perfusion actually alter tissue physiology in hybrid systems.

(b) Mechanical sensing and readouts

Morales and co-workers reviewed microfluidic-compatible methods to measure tissue traction, stiffness, and contractility on-chip, arguing that mechanical readouts are often the most sensitive reporters of maturation and disease phenotype. Integrating such mechanical sensors with biofabricated constructs therefore provides a non-invasive route to quantify functional maturation post-printing [51].

Sensitive mechanical readouts are now embedded directly into microphysiological devices. In 2020, Kim and colleagues engineered a polydimethylsiloxane (PDMS)-encapsulated crack sensor on silicone cantilevers to quantify cardiomyocyte contractility continuously for more than 26 days in culture, enabling drug-response and maturation assays based on force rather than only electrophysiology. This approach shows that mechanical sensors can report functional maturation of printed cardiac constructs without destructive sampling [67].

Complementing crack sensors, in 2023 Wu et al. generated a model for instrumented hybrid printed-chip constructs by integrating iPSC-derived cardiac tissues with embedded electrical and mechanical sensors, enabling simultaneous measurement of electrophysiology and contractile force under perfusion and pacing. Specifically, they printed vertical poly(3,4-ethylenedioxythiophene): polystyrene sulfonate (PEDOT:PSS) micropillars and elastic microwires as integrated microelectrodes and nanocomposite force sensors in a heart-on-a-chip, demonstrating simultaneous electrical and mechanical monitoring of iPSC-cardiac tissues [68]. Such platforms provide functional benchmarks that validate whether engineered perfusion and structural cues produce clinically relevant tissue behaviour.

(c) Sensor integration for real-time readouts

To move beyond endpoint assays, chips increasingly embed electrical, optical, and electrochemical sensors for continuous monitoring of barrier integrity, metabolism, pH, and electrophysiology. Chen et al. reviewed sensor modalities integrated with OoCs and showed multiple examples where on-chip sensors provided earlier, more sensitive readouts of tissue state than conventional assays [69].

Research groups have moved from off-chip endpoint assays to in-line sensors for transepithelial/transendothelial electrical resistance (TEER), oxygen, and metabolites. Lucchetti et al. patterned in 2024 flexible, thin-film electrodes for spatially resolved TEER in complex chip geometries, enabling continuous mapping of barrier integrity across channels, a capability that validates whether printed vasculature and perfusion preserve endothelial barrier function [70].

In 2023 Marrero et al. implemented semi-transparent PEDOT:PSS electrodes for impedance spectroscopy of epithelial barriers on-chip, showing improved signal coupling and compatibility with optical imaging; such electrodes make it practical to couple printed tissues to real-time electrical readouts without occluding microscopy [71].

(d) Bioprinting and chip manufacturing

Manufacturing perspectives now emphasise printing of microfluidic features and direct deposition of cell-laden inks into chip cavities, improving registration between printed vasculature and perfusion ports. Chliara et al. [72] and other manufacturing reviews [73] highlight workflows that combine bioprinting and microfabrication to produce chip-compatible constructs at scale.

Practical workflows now combine 3D printing of electronics/microelectrodes with deposition of cell-laden inks into chip cavities. Lind et al. [24] and more recent work by Wu et al. [68] demonstrate that multi-material printing can embed strain gauges, conductive traces, and microelectrodes within a microdevice while simultaneously patterning tissues, enabling “one-shot” manufacturing of instrumented hybrid platforms that minimise manual assembly and registration errors.

Reviews and manufacturing studies (2023–2024) also show practical recipes for printing perfusable ports and aligning printed vasculature with chip fluidics, which reduces handling damage to delicate printed or organoid aggregates and facilitates standardised interfacing [74,75].

(e) Multi-tissue coupling and recirculating vascular flow

OoC systems have progressed toward linking matured tissue niches via recirculating vascular loops to model inter-organ communication and pharmacokinetics [18]. The broader OoC literature documents how recirculating endothelialised channels allow preclinical testing of biofabricated constructs by preserving tissue phenotypes and enabling physiologically relevant cross-talk [15].

In 2022 Ronaldson-Bouchard et al. built a multi-organ chip connecting matured heart, liver, bone, and skin niches by recirculating endothelialised vascular flow and showed preserved adult-like function and physiologic crosstalk over extended culture. This study supplies a practical blueprint for testing printed, vascularised tissues in a systemic context (drug PK/PD, inter-organ signalling) rather than as isolated constructs [52].

Maggiore et al. further show that engineered endothelial niches, generated by genetically inducible endothelial programmes, can vascularise kidney organoids and drive multilineage maturation; this is an example of combining biological endothelial programming with engineered perfusion to produce mature hybrid tissues on-chip [57]. The limitations of conventional organ-on-chip platforms and hybrid strategies, developed from 2020 to 2025, enabling complex tissue integration, are presented in Table 5.

#### 3.2.3. Practical Impact and Unresolved Technological Gaps

**Figure 2:**
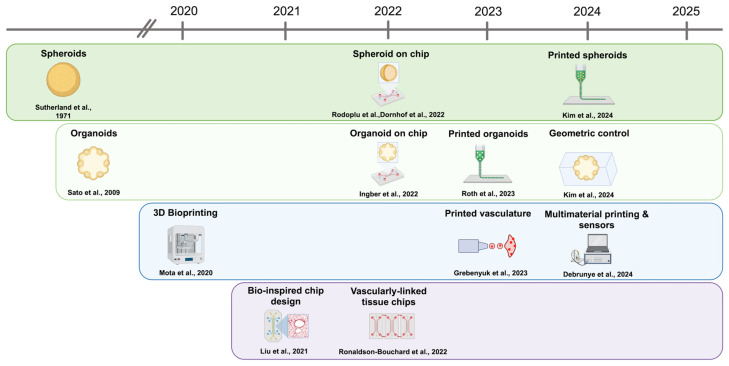
Evolution of in vitro tissue platforms toward hybrid biofabrication. Foundational spheroid (dark green), organoid (light green), bioprinting (blue), and organ-on-chip platforms (purple) initially developed as standalone systems have progressively converged toward hybrid architectures. Integration of biological self-organisation with engineered spatial control, perfusion, and instrumentation has enabled increasingly complex and functional tissue models. The information depicted has been extracted from the following original studies: [4,7,18,23,26,29,34,35,37,43,52,56]. Created in BioRender.

When combined with biofabricated scaffolds, OoCs provide the controlled perfusion, mechanical conditioning, and continuous sensing required to validate and mature printed, spheroid-based, or organoid-laden constructs. Quintard et al. [33] and related organoid-chip studies [49] show outcomes essential for disease modelling and drug testing, such as improved oxygenation, longevity and function. These trends reflect a broader convergence of biofabrication technologies toward hybrid systems (Figure 2).

However, many challenges still persist. Standardised interfaces between printed constructs and chips are missing, many sensor modalities remain difficult to miniaturise without interfering with tissue microenvironments, and throughput/standardisation for preclinical pipelines is still limited. Addressing these engineering bottlenecks through modular chip geometries, standardised perfusion ports, and sensor-friendly materials will be crucial to deploying hybrid biofabricated tissues at scale. These themes lead naturally into Section 4, where we discuss specific hybrid strategies that merge the architectural precision of bioprinting with the dynamic control of organ-on-chip platforms.

## 4. Hybrid Strategies: Bridging Engineered Control and Biological Self-Organisation

**Figure 3:**
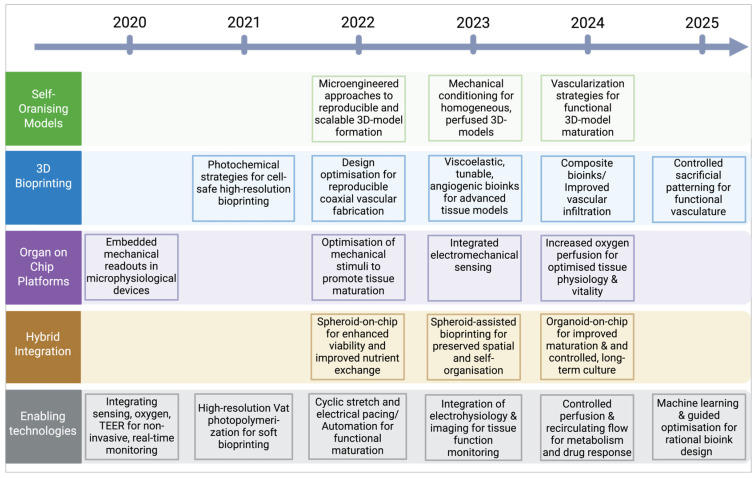
Summary of technological advancements of in vitro biofabricated models from 2020 to 2025. This timeline summarises the major technological developments reported during 2020–2025 across the major biofabrication systems. Additionally, the table highlights the drivers of the field’s evolution, highlighting the emerging technologies that enabled the transition from one innovation to the next. The information depicted has been collected by the original studies mentioned in the review: [27,28,29,30,31,32,33,34,35,36,37,38,39,40,41,42,43,44,45,46,47,48,49,50,51,52,53]. Created in BioRender.

Hybrid biofabrication intentionally couples engineered structure, perfusion, and instrumentation with the intrinsic morphogenetic programmes of organoids and spheroids. The last five years (2020–2025) produced concrete demonstrations that (i) engineered boundary conditions can direct organoid patterning without abolishing self-organisation, (ii) engineered perfusion both relieves diffusion limits and synergises with biological angiogenesis, and (iii) embedded sensing and computational design enable iterative, data-driven optimisation of hybrid constructs. A consolidated overview of the key technological milestones and the drivers underpinning this transition across biofabrication platforms between 2020 and 2025 is provided in Figure 3. Together, these advances define hybrid biofabrication as a design paradigm in which technical constraints guide, but do not prevail over, biological self-organisation.

### 4.1. Engineering Boundary Conditions That Shape Morphogenesis

Geometrical and transport constraints, such as boundary curvature, nutrient flux, and confinement, can steer developmental programmes with high reproducibility. The UniMat platform established a 3D geometrically engineered permeable-membrane array that produces uniform organoids with accelerated maturation by controlling nutrient flux and boundary curvature. The study shows how relatively simple engineered boundaries reduce morphological variance while preserving organoid differentiation programmes [39]. Complementary work used lithographically defined microchambers to bias brain-organoid axis formation and cortical lamination, demonstrating that physical constraints can substitute for stochastic symmetry breaking while preserving cell polarity and regional identity [76].

### 4.2. Engineered Perfusion That Complements Biological Vascularisation

Directly supplying flow via printed or chip-integrated channels relieves hypoxia and accelerates functional maturation. Quintard et al. embedded diverse 3D tissues adjacent to endothelialised microchannels on a user-friendly chip and reported sustained intravascular perfusion for weeks, together with improved oxygenation and organoid function, compared with static culture [33]. For quantitative validation, real-time oxygen mapping using NIR ratiometric nanosensors has allowed groups to directly correlate channel perfusion with reduced hypoxic cores and improved metabolic profiles inside spheroids and organoids, giving a rigorous metric to evaluate hybrid vascular strategies that combine immediate convective transport with longer-term biological vessel formation [77,78].

### 4.3. Mechanical and Biochemical Feedback Loops to Accelerate Maturation

Mechanical stimulation delivered by engineered systems can markedly accelerate tissue maturation. Multi-tissue circuits with recirculating vascular flow demonstrated that matured heart, liver, bone, and skin niches preserve adult-like phenotypes and physiologic cross-talk over extended culture, showing that systemic recirculation and organ–organ signalling are reachable in hybrid setups [52]. In addition, studies that apply cyclic strain or pressure to printed constructs report alignment, matrix remodelling, and enhanced parenchymal function. These results indicate that artificial mechanical signals can be used to “train” self-organised tissues to achieve performance similar to that of adult tissues, often at a faster timescale than biochemical signals alone [79].

### 4.4. Embedded Sensing and Instrumented Hybrids for Continuous Readouts

Instrumented hybrid systems provide the continuous, non-destructive readouts required for optimisation. Recent work integrates thin-film TEER electrodes, microelectrode arrays, and optical/metabolic sensors into chip and printed scaffolds, enabling real-time monitoring of barrier integrity, electrophysiology, and metabolism during maturation and drug testing [80]. For example, microfabricated, on-chip TEER and optical sensors have been shown to detect barrier disruption and metabolic shifts earlier than endpoint assays, which is critical for validating whether engineered perfusion and structural cues produce functional gains [80,81].

### 4.5. Computational and Closed-Loop Optimisation of Hybrid Systems

**Figure 4:**
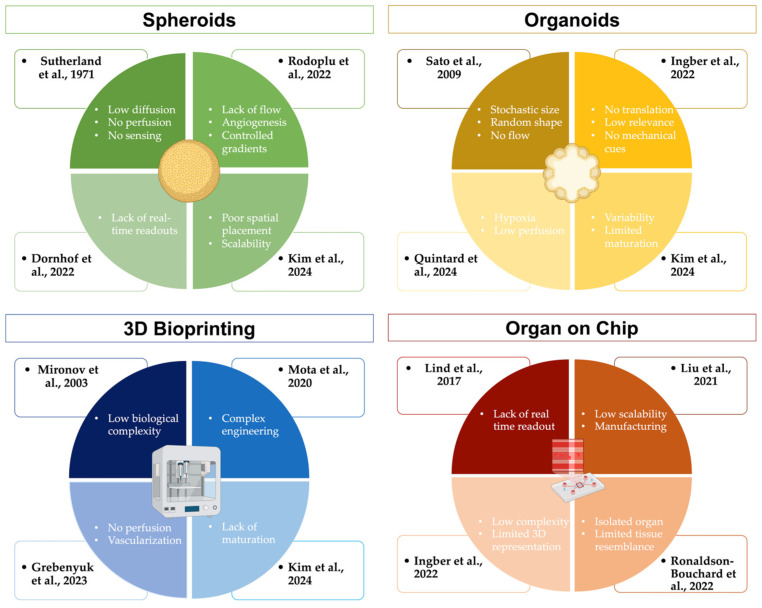
Drivers of hybrid biofabrication and key boundary conditions addressed by recent strategies. The schematic illustrates how engineered spatial boundaries, vascularisation/perfusion, mechanical cues, sensing and feedback, and multimodal hybrid assembly interact with intrinsic self-organisation principles to enable functional and predictive tissue models. Representative strategies and mechanisms from recent literature are mapped to each driver. Spheroids are shown in green, organoids in yellow, 3D bioprinting in blue and organ-on-chip strategies in brown. The information depicted has been extracted from the following original studies: [4,7,18,21,23,24,26,29,33,34,37,43,52]. Created in BioRender.

As hybrid biofabrication involves numerous interdependent variables, such as bioink composition, printing parameters, architecture, and culture conditions, data-driven and feedback-controlled approaches are increasingly essential. For example, Limon et al. developed a machine-learning model predicting filament width from print parameters and bioink composition with ~85% accuracy in extrusion-based bioprinting, highlighting how ML can reduce empirical trial-and-error in scaffold architecture [53]. In another study, Sarah et al. trained random forest models to predict hybrid hydrogel rheology from composition and shear rates (R^2^ ≈ 0.99), showing how formulation optimisation can be automated [82]. On the sensing side, Chen et al. integrated organoid-biosensor interfaces for continuous monitoring of physiological parameters, indicating that real-time embedded sensing is feasible in complex, living constructs [83]. Together, these drivers define hybrid biofabrication as a closed-loop design paradigm in which engineered boundary conditions guide, but do not replace, intrinsic self-organisation; this conceptual framework and its key enabling mechanisms are summarised in Figure 4 and exemplified by representative hybrid strategies in Table 6.

The key technological innovations that enabled the emergence of hybrid biofabrication paradigms, together with their impact on tissue modelling and representative studies across major biofabrication systems, are summarised in Table 7.

## 5. Key Gaps and Translational Hurdles

Despite compelling proof-of-principle demonstrations, hybrid biofabrication faces several interdependent barriers that limit reproducibility, functional maturity, and regulatory readiness for industrial and clinical translation. Below, we summarise the experimentally unaddressed gaps and why they matter for translating hybrid biofabricated systems.

### 5.1. Standardisation and Reproducibility

Hybrid workflows layer multiple fabrication steps, such as organoid growth, bioink formulation, printing, and chip assembly. Each of these parameters is a source of variability. Sockell et al. developed an automated microwell platform that quantifies morphological variance across hundreds of organoids and showed that image-based quality control (QC) predicts downstream differentiation outcomes, underscoring how automated, quantitative gating can reduce batch-to-batch variation [41].

Conversely, cross-platform surveys and method comparisons reveal that small differences in device geometry and material stiffness materially change outcomes, meaning that without open hardware designs and validated materials libraries, results remain difficult to replicate across laboratories [42].

### 5.2. Functional Maturation

Engineered perfusion and mechanical conditioning often improve morphology and short-term function but do not always drive adult-like transcriptional programmes. Maggiore et al. induced endothelial niches to vascularise kidney organoids and observed improved nephron organisation and renin-expressing cells, but persistent foetal gene expression remained, showing that vascularisation alone is insufficient to fully mature organoids [57]. Similarly, studies of neural assembloids reveal robust regional patterning yet limited long-term synaptic maturity without prolonged conditioning or additional cues [84]. These results highlight a recurring discrepancy between structural or functional improvements and the acquisition of adult-like tissue molecular identity.

### 5.3. Multi-Compartment Integration: Immune, Neural, and Endocrine Axes

A further translational gap is integrating missing physiological axes. Self-organising neuromuscular models (e.g., Urzi et al.) show that neuromuscular junctions can form in vitro, but stability and long-term function often degrade without optimised media and synchronised maturation schedules [85]. Co-culture studies therefore demonstrate feasibility but also expose fragility. Stable, multi-lineage integration demands matched timelines, compatible media, and dynamic perfusion that maintains gradients of cytokines and neurotransmitters.

### 5.4. Materials, Sensors, and Long-Term Device Stability

Hybrid systems combine hydrogels, elastomers, and electronics that age differently. Debruyne et al. used NIR oxygen nanosensors to quantify how perfusion reduces hypoxic cores in spheroids, an example of the value of embedded sensors, but sensor performance and material compatibility remain concerns as cultures extend from days to weeks or months [35].

Device sensor drift and mechanical fatigue have been documented in PDMS and printed elastomer systems, where repeated actuation or swelling alters calibration and signal fidelity over weeks. Experimental reports of strain-sensor fabrication and on-chip fatigue highlight the need for sensor-grade materials and routine recalibration protocols [86].

### 5.5. Pathways to Regulatory and Ethical Approval

Finally, regulatory acceptance requires standardised evidence of assay performance. Ingber and others have argued that reproducible, mechanistically interpretable readouts and cross-lab validation studies are prerequisites for pharmaceutical qualification of OoC/hybrid assays [18].

Ethical questions (patient-derived cells, brain-organoid complexity) also demand transparent governance frameworks and standard reporting of provenance and differentiation state. Together, regulatory and ethical considerations highlight the need for standardised reference criteria that link device performance, biological fidelity and the intended context of use.

A consolidated overview of the remaining technological and biological challenges across major biofabrication systems, together with their underlying causes and representative solution strategies, is summarised in Table 8.

## 6. Future Prospects and Outlook

Looking forward, the convergence of biological self-organisation with engineered control is expected to further transform in vitro tissue modelling [25,34]. Hybrid biofabrication strategies that combine organoids or spheroids with defined geometry, perfusable vasculature, and dynamic mechanical conditioning are likely to become the dominant paradigm for achieving both physiological relevance and experimental reproducibility [34].

In the near term, advances in standardised bioinks, modular printing strategies, and chip-compatible interfaces will facilitate cross-laboratory reproducibility and scalability [87,88]. Integration of real-time sensing and closed-loop control is expected to accelerate tissue maturation by enabling adaptive tuning of perfusion, mechanical stimulation, and biochemical cues [73].

In the longer term, coupling hybrid biofabricated tissues into multi-organ microphysiological systems, together with data-driven optimisation and machine-learning-guided design, may enable predictive platforms for drug development and disease modelling. Addressing remaining challenges in standardisation, regulatory acceptance, and long-term stability will be essential to translate these systems from experimental tools into robust preclinical and industrial platforms.

## 7. Conclusions

Hybrid biofabrication is redefining how we model, study, and eventually reconstruct living tissues. By merging the self-organising capacity of stem cell-derived systems, such as spheroids and organoids, with the spatial precision and dynamic control of tissue biofabrication, including 3D bioprinting and organ-on-chip technologies, the field has moved beyond proof-of-concept demonstrations toward physiologically faithful, perfusable, and scalable model systems. Over the past five years (2020–2025), progress in vascularised organoids, hybrid bioinks, and instrumented microphysiological platforms has shown that integration, rather than substitution, between biological and engineering paradigms is the key to achieving reproducibility and functional maturity.

Yet, many critical challenges remain unaddressed. These include standardising hybrid workflows across laboratories, aligning mechanical and biological timescales of tissue maturation, and ensuring the long-term stability of integrated materials and sensors. Addressing these gaps will require open-source design standards, quantitative benchmarking, and data-driven feedback loops that close the gap between fabrication parameters and biological outcomes. Hybrid biofabrication thus represents not merely a technical synthesis but a conceptual framework for engineering with, rather than against, the intrinsic rules of developmental biology.

## Figures and Tables

**Table 1:** Limitations of conventional spheroids and hybrid strategies developed to overcome them (2020–2025). This table summarises the principal structural, transport, and functional limitations of stand-alone spheroid models; the hybridisation strategies (microfluidic perfusion, vascular interfaces, sensor integration, and bioprinting-assisted positioning) introduced to address these constraints; and the resulting improvements in oxygenation, metabolic stability, and functional readouts. Representative studies illustrate how spheroids are transitioning from static aggregates to modular building blocks for hybrid tissue systems.

System	Boundary/Limitation	Strategy/Hybridisation	Key High-Impact Studies
Spheroids	Diffusion-limited No perfusion No sensing	Self-assembled multicellular aggreggates	Sutherland et al., 1971 [4]
Spheroid-on-Chip	Lack of flow No angiogenesis Controlled gradients	Microfluidic confinement Extended perfusion	Rodoplu et al., 2022 [29]
Spheroid-on-Chip (instrumented)	No real-time functional readout	Integrated sensors (O_2_, electrodes)	Dornhof et al., 2022 [26]
Printed Spheroids	Poor spatial placement Limited scalability	High-throughput bioprinting of spheroids	Kim et al., 2024 [34]

**Table 2:** Limitations of conventional organoid cultures and hybrid engineering strategies enabling control and maturation (2020–2025). The table outlines key bottlenecks of stand-alone organoids, including diffusion limits, morphological variability, and lack of mechanical and vascular cues, together with hybrid approaches such as geometrical confinement, perfusion-on-chip, vascular co-culture, and automated phenotyping. The functional outcomes demonstrate how guided self-organisation enhances reproducibility, maturation, and physiological relevance. Representative studies are listed for each strategy.

System	Boundary/Limitation	Strategy/Hybridisation	Key High-Impact Studies
Organoids	Stochastic size Random shape Limited flow	Self-organization from stem cells	Sato et al., 2009 [7]
Engineered Confinement	Variability Limited maturation	Geometric boundary control	Kim et al., 2024 [34]
Organoid-on-Chip	Hypoxia No perfusion	Microfluidic perfusion Vascular coupling	Quintard et al., 2024 [33]
Organoid-on-Chip (conceptual)	Limited translational potential Low physiological relevance	Organs-on-chip paradigm	Ingber et al., 2022 [18]
Printed Organoid Assemblies	Poor spatial organization	Bioprinted assembloids	Roth et al., 2023 [56]

**Table 3:** Limitations of stand-alone bioprinting and hybrid strategies integrating biological self-organisation (2020–2025). This table summarises the main constraints of purely printed tissues, including limited biological complexity, immature phenotypes, and insufficient microvascular resolution, and highlights hybrid solutions such as spheroid- or organoid-assisted printing, sacrificial vascular templating, coaxial extrusion, and dynamic perfusion. Functional outcomes illustrate how hybrid biofabrication enhances tissue maturation, perfusion, and stability. Representative studies are provided.

System	Boundary/Limitation	Strategy/Hybridisation	Key High-Impact Studies
Bioprinting	Limited biological complexity	Layer-by-layer tissue printing	Mironov et al., 2003 [21]
Bioprinting (Review Baseline)	Engineering-heavy Biologically poor	Biofabrication principles	Mota et al., 2020 [23]
Perfused printed Tissues	No perfusion	Soft microfluidics Printed vasculature	Grebenyuk et al., 2023 [43]
Printed self-organized Tissues	Lack of maturation	Printing spheroids/organoids	Kim et al., 2024 [34]

**Table 4:** Comparative strengths of biofabrication systems. Comparison of 3D culture and biofabrication approaches mentioned in the review across various properties. “+” symbols indicate relative strength, as follows: + = Limited capability; ++ = Moderate capability; +++ = High capability. Categorisation and grading are derived from the original studies: [25,29,30,32,33,34,41,45,46,52,56,67,68].

Category	Biological Self-Organisation & Cellular Complexity	Architectural & Spatial Control	Perfusion & Vascular Integration	Mechanical & Electrical Conditioning	Physiological Monitoring & Readouts	Scalability & Reproducibility
System						
Spheroids	++	+	+	+	+	+
Organoids	+++	+	+	+	+	+
3D Bioprinting	+	+++	++	++	++	++
Organ-on-Chip Platforms	+	++	+++	+++	+++	+++
Hybrid Approaches	+++	+++	+++	+++	+++	+++

**Table 5:** Limitations of conventional organ-on-chip platforms and hybrid strategies enabling complex tissue integration (2020–2025). The table presents major constraints of stand-alone OoC systems, such as limited tissue complexity, absence of self-organisation, and restricted long-term maturation, together with hybrid solutions including bioprinted tissues, organoid integration, engineered vascular networks, and embedded sensing. The listed functional outcomes show how hybridisation improves tissue longevity, physiological fidelity, and translational relevance.

System	Boundary/Limitation	Strategy/Hybridisation	Key High-Impact Studies
Organ on Chip	Limited 3D tissue complexity	Microfluidic physiological control	Ingber et al., 2022 [18]
OoC Foundations	Scaling & manufacturability	Bioinspired chip design	Liu et al., 2021 [37]
Instrumented OoC	Lack of real-time readouts	Multimaterial printing Sensors	Lind et al., 2017 [24]
Multi-Organ Hybrid OoC	Organ-isolated physiology	Vascularly linked tissue chips	Ronaldson-Bouchard et al., 2022 [52]

**Table 6:** Key hybrid biofabrication strategies: limitations, engineering solutions, and functional outcomes (2020–2025). This table summarises representative hybrid biofabrication studies, identifying (i) the primary limitation of stand-alone biological or engineered systems, (ii) the hybrid strategy implemented to overcome this constraint (e.g., perfusion, geometrical confinement, vascularisation, sensing, or modular assembly), and (iii) the resulting functional or translational improvement. Together, the examples illustrate how hybridisation bridges the gap between biological self-organisation and engineered control.

Publication	Boundary/Limitation	Proposed Strategy
Bioprinting of Cells, Organoids and Organs-on-a-Chip Together with Hydrogels (2024)	Organoid cultures lack spatial control, mechanical cues and integrative architecture; static conditions limit mechanobiological insights.	3D/4D bioprinting with organoids and organ-on-chip using engineered hydrogels; scaffolds for defined mechanical and structural cues and combinatorial control of physical and biochemical microenvironments.
Organoid bioprinting: from cells to functional tissues (2024)	Conventional organoid self-organization lacks predetermined 3D architecture and scalable patterning.	Use of continuous and pick-and-place bioprinting methods to position spheroids/organoid-forming cells precisely, enabling hierarchical and reproducible tissue assembly while preserving biological self-organization
A microfluidic platform integrating functional vascularized organoids-on-chip (2025)	Organoids on chips commonly lack functional vasculature and realistic perfusion.	Combined vascular network formation within microfluidic chips that connect endothelial networks to spheroids and organoids, enabling intravascular perfusion and sustained culture.
Advances in 3D Bioprinting and Microfluidics for Organ-on-Chip Platforms (2025)	Organ-on-chip platforms often miss dynamic physiological control and scalable fabrication.	Convergence of high-resolution 3D bioprinting with microfluidic design enables precise spatial control of tissues and fluids, embedded sensors, and multi-organ connectivity.
Converging bioprinting and organoids to better recapitulate the tumor microenvironment (2024)	Tumor organoid models fail to mimic complex microenvironments and lack vascular perfusion.	Hybrid fabrication combining 3D bioprinting of tumor organoids with organ-on-chip components to introduce perfusion and structural heterogeneity reflective of the tumor microenvironment.
Recent advances in biofabrication strategies based on bioprinting for vascularized tissue repair and regeneration (2023)	Bioprinted constructs lack mature, perfusable vasculature at multiple scales.	Use of multi-scale bioinks, growth factor patterning, and printing strategies that encourage vessel formation and tissue integration.
Bioengineering methods for vascularizing organoids (2024)	Organoids suffer from hypoxia, limited nutrient transport and lack of intrinsic vasculature.	Vascularization via co-culture of vascular cells/organoids, co-differentiation, organoid-on-a-chip integration, and 3D bioprinting for perfusable features.

**Table 7:** Key technological innovations enabling the emergence of hybrid biofabrication paradigms and their impact on tissue modelling. The table summarises major advances in the fields of spheroids, organoids, 3D bioprinting, organs-on-a-chip and hybrid systems, highlights their functional impact on reproducibility, maturation and physiological relevance, and lists representative studies illustrating each advance.

System	Key Advances	Impact on Field	Representative Studies
Spheroids	Microengineered spheroid formation using hanging-drop microfluidics Droplet hydrogels Optimized microwells	Reproducible Homogeneous spheroids with enhanced viability Reduced batch variation	Rodoplu 2022 [29]; Khan 2022 [30]; Wongpakham 2024 [31]
Organoids	Geometric confinement High-throughput microwell platforms Controlled organoid size and maturation	Reduced variability Improved phenotypic consistency Disease modeling and screening	Kim 2024 (UniMat) [34]; Sockell 2023 [41]
3D Bioprinting	Engineered vascular networks through coaxial extrusion Sacrificial templating Lumen-stabilizing bioinks	More perfusable Scalable printed tissues Improved nutrient delivery	Malkani 2025 [45]; Bosch-Rué 2022 [46]
Organ-on-Chip platforms	Integrated perfusion and multi-modal sensing Continuous real-time tissue monitoring	Stable long-term cultures Dynamic physiological readouts	Fang 2023 [32]; Kim 2020 [67]; Wu 2023 [68]
Hybrid Approaches	Convergent constructs Vascularization Fluid flow 3D printing	Enhanced maturation Sustained perfusion Systemic-level interactions	Quintard 2024 [33]; Roth 2023 [56] Huang 2025 [25]; Ronaldson-Bouchard 2022 [52]

**Table 8:** Remaining challenges, Underlying causes, and Emerging solution strategies for major biofabrication platforms. The table summarises the main biological and technological limitations associated with spheroids, organoids, 3D bioprinting, organ-on-a-chip systems and hybrid approaches, links these challenges to their main underlying causes, and summarises representative strategies proposed to address them. Selected studies illustrating each category are provided.

System	Remaining Challenges	Underlying Causes	Promising Solutions	Representative Studies
Spheroids	Long-term maturation Diffusion limits Weak architectural control	No intrinsic vasculature Static culture Reliance on passive self-assembly	Microfluidic perfusion Vascularized encapsulation Spheroid-assisted biofabrication	Fang 2023 [32]; Quintard 2024 [33]; Roth 2023 [56]
Organoids	Persistent heterogeneity Restricted size Insufficient mechanical cues	Probabilistic self-organization Absence perfusion and dynamic strain	Constrained microenvironments Organoid-on-chip perfusion Pattern-guided fabrication	Huang 2025 [25]; Quintard 2024 [33]; Kim 2024 (UniMat) [34]
3D Bioprinting	Immature vasculature Loss of function over time Thick-tissue hypoxia	Printed channels lack adaptive remodeling Mechanical mismatch Low oxygen tension	Hybrid vascularization Sacrificial/embedded printing Growth-factor patterning	Huang 2025 [25]; Malkani 2025 [45]; Bosch-Rué 2022 [46]
Organ-on-Chip platforms	Limited biological complexity Challenges scaling to multi-organ integration	Limited biological complexity Challenges scaling to multi-organ integration	Multi-tissue platforms Soft biomaterials Multimodal sensors	Ronaldson-Bouchard 2022 [52]; Wu 2023 [68]
Hybrid Approaches	Integration complexity Reproducibility High engineering overhead	Need to coordinate self-assembly with engineered constraint Complex multi-physics	Standardized vascular templates Guided self-organization Adaptive perfusion circuits	Huang 2025 [25]; Quintard 2024 [33]; Malkani 2025 [45]

## Data Availability

The original contributions presented in this study are included in the article. Further inquiries can be directed to the corresponding authors.
